# Impact of skeletal muscle mass in patients with recurrent gastric cancer

**DOI:** 10.1186/s12957-021-02283-6

**Published:** 2021-06-11

**Authors:** Tomoyuki Matsunaga, Hiroaki Satio, Wataru Miyauchi, Yuji Shishido, Kozo Miyatani, Yuki Murakami, Takehiko Hanaki, Kyoichi Kihara, Manabu Yamamoto, Naruo Tokuyasu, Shuichi Takano, Teruhisa Sakamoto, Toshimichi Hasegawa, Yoshiyuki Fujiwara

**Affiliations:** 1 Division of Gastrointestinal and Pediatric Surgery, Department of Surgery, School of Medicine, Tottori University Faculty of Medicine, 36-1 Nishi-cho, Yonago, 683-8504 Japan; 2 Department of Surgery, Japanese Red Cross Tottori Hospital, 117 Shotoku-cho, Tottori, 680-8517 Japan

**Keywords:** Gastric cancer, Chemotherapy, Skeletal muscle mass

## Abstract

**Background:**

We retrospectively examined the relationship among skeletal muscle mass index (SMI), prognosis, and chemotherapy side effects in patients with recurrent gastric cancer (RGC).

**Methods:**

Sixty-seven patients who developed recurrence after undergoing curative gastrectomy for gastric cancer at Tottori University Hospital and received palliative chemotherapy were included in this study. Pretreatment computed tomography was performed to measure the skeletal muscle mass (SMM) and cross-sectional SMM at the third lumbar vertebra. We focused on haematologic toxicity (neutropenia, thrombocytopenia, and anaemia), febrile neutropenia, and gastrointestinal toxicity (diarrhoea, vomiting, and stomatitis) as the side effects of chemotherapy.

**Results:**

Median SMIs for males and females (43.9 and 34.7 cm^2^/m^2^, respectively) were used as cutoff values. The patients were classified into high (SMI^High^; *n* = 34) and low SMI groups (SMI^Low^; *n* = 33). The SMI^Low^ group included more patients treated with monotherapy (*P* = 0.016) compared with the SMI^High^ group, had a significantly lower number of chemotherapy lines (*P* = 0.049), and had a significantly higher incidence of grade 3 or 4 side effects (*P* = 0.010). The median survival rate was significantly higher in the SMI^High^ group (17.8 vs 15.8 months; *P* = 0.034). In the univariate analysis, body mass index, SMI, histological type, and prognostic nutritional index were identified as prognostic indicators. The multivariate analysis identified SMI (*P* = 0.037) and histological type (*P* = 0.028) as independent prognostic factors.

**Conclusion:**

The incidence of grade 3 or 4 side effects was significantly higher in patients with SMI^Low^ RGC. SMI was a useful prognostic marker of RGC.

## Introduction

Gastric cancer remains the fourth most common cancer and the second leading cause of cancer-related death worldwide [1, 2]. Gastrectomy is the main treatment strategy for gastric cancer. However, patients with advanced gastric cancer often experience recurrence [3, 4]. Chemotherapy is the main treatment for recurrent gastric cancer (RGC). Despite the improvements in prognosis as well as survival outcomes in patients with RGC, the overall outcome remains poor [5, 6].

Sarcopenia is characterised by a loss of skeletal muscle mass (SMM) and has been widely reported to impair physical performance and survival in the elderly [7, 8]. The relationship between sarcopenia and prognosis has been reported in various cancers, including gastric cancer [9–17]. In patients with cancer, sarcopenia is more likely to develop due to increased protein catabolism, inflammatory reactions, metabolic abnormalities, and poor oral intake. Sarcopenia may be associated with cancer cachexia [18]. Cancer cachexia disturbs the regenerative ability of skeletal muscle [19]. Patients with advanced gastric cancer often receive perioperative chemotherapy and the side effects of chemotherapy can cause loss of SMM. In addition, patients experience weight loss after gastrectomy because of poor dietary intake, which leads to various postoperative disorders and SMM loss [20, 21].

At the time of recurrence after gastrectomy, treatment options are limited to chemotherapy or best supportive care, and chemotherapy is administered despite the decrease in SMM. Sarcopenia is reported to possibly influence the pharmacokinetics of chemotherapy, which could be associated with the adverse effects of chemotherapy in several cancers [22]. However, there are few reports on SMM and chemotherapy side effects in patients with RGC. Moreover, there are few reports on SMM and RGC prognosis at the time of recurrence.

In this study, we retrospectively examined the relationship among skeletal muscle index (SMI), prognosis, and side effects of chemotherapy in patients with RGC after undergoing gastrectomy.

## Patients and methods

### Patients

Between January 2008 and December 2019, 605 patients were pathologically diagnosed with gastric cancer and had undergone curative gastrectomy at Tottori University Hospital. Gastrectomy was performed with D2 lymph node dissection for advanced cancer and D1+ lymph node dissection for early gastric cancer. Patients with stage II or III gastric cancer underwent adjuvant chemotherapy according to the Japanese gastric cancer treatment guidelines [23]. Sixty-seven patients developed recurrence, received palliative chemotherapy, and were included in this study. All the patients received first-line chemotherapy 4 weeks after a computed tomography (CT) scan. Clinicopathological findings were determined according to the Japanese gastric cancer treatment guidelines [23]. Clinical data, including age, sex, histology, history of gastrectomy, and metastatic site at the time of recurrence, were collected from electronic medical records. The patients were followed up every 3 months to check for recurrence by performing blood tests, including those for tumour markers, and by physical examination after the operation. Moreover, CT was performed at least every 6 months after the operation. Recurrence patterns and causes of death were examined from clinical records, CT, and positron emission tomography–CT. A family inquiry was conducted for patients who were difficult to follow up.

### Definition of SMI

**Fig. 1:**
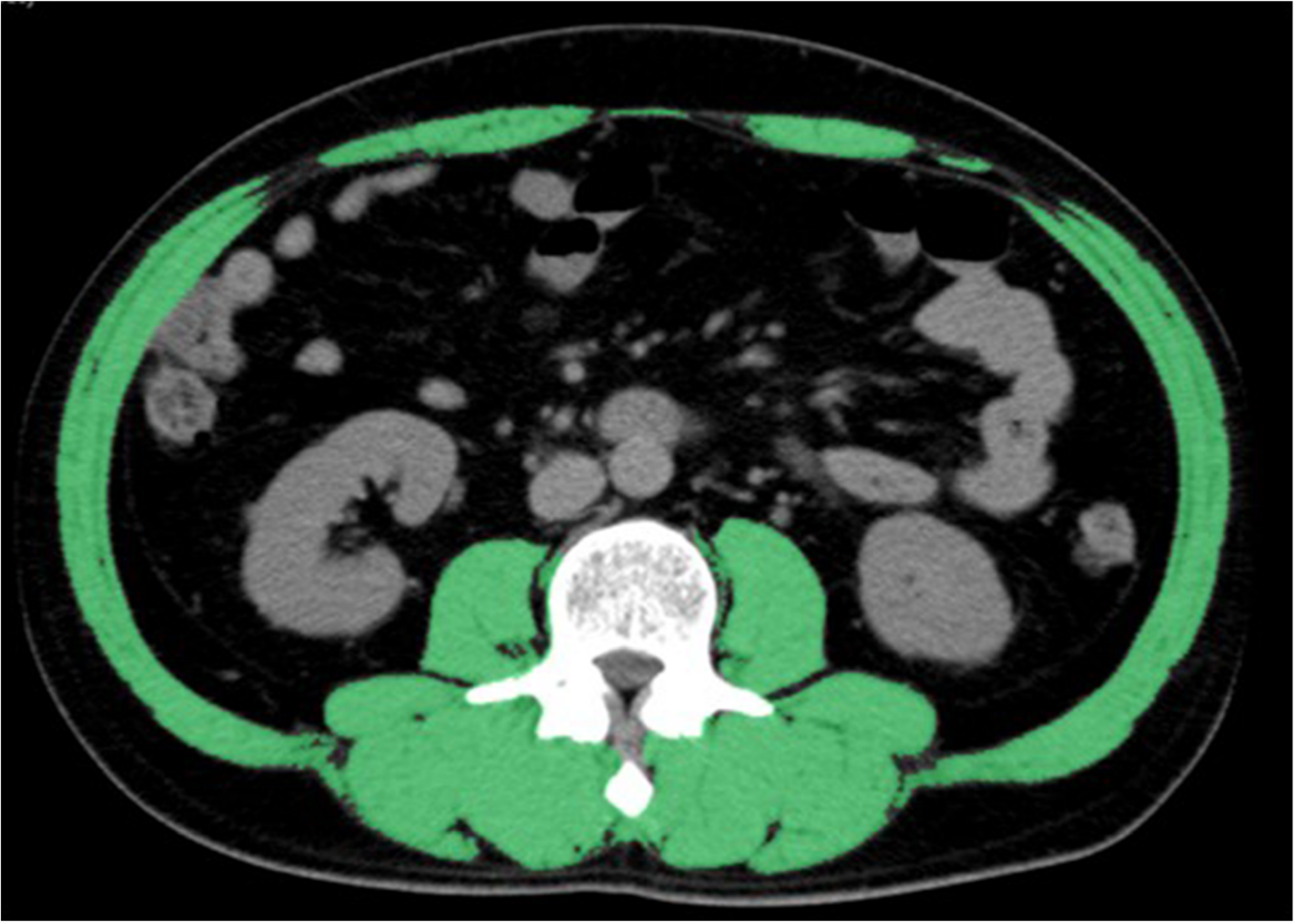
Measurement of SMM in RGC patients. Axial computed tomography slice of the third lumbar vertebra (L3). Green areas indicate skeletal muscle mass. Abbreviations: RGC, recurrent gastric cancer; SMM, skeletal muscle mass

Pretreatment CT was performed to measure SMM and the SYNAPSE VINCENT system was used to measure the cross-sectional SMM at the level of the third lumbar vertebra (L3) [24]. The areas covered by SMM were calculated from pixels in the density range of − 29 to + 150 Hounsfield Units [25], which includes muscle and intra-abdominal organs but excludes bone and fat. The L3 region comprises the psoas, paraspinal, and abdominal wall muscles (Fig. 1). The skeletal muscle area in a single abdominal image is proportional to the whole-body muscle mass [26], and SMI is defined as the muscle area normalised by the square of the height (m^2^) [27].

### Details of first-line chemotherapy

The standard first-line palliative systemic chemotherapy was a fluoropyrimidine-, taxane-, or irinotecan-based regimen that was administered in accordance with the gastric cancer treatment guidelines for each decade [23, 28]. At the physician’s discretion, a single agent was used in patients with PS 2, those older than 80 years, or those who refused combined chemotherapy. In this study, monotherapy was administered in 23 patients and combination chemotherapy was administered in 44 patients. The monotherapy regimens included the following: (i) CPT-11 (*n* = 11), (ii) S-1 (*n* = 8), and (iii) paclitaxel (*n* = 4). The combination chemotherapy regimens were as follows: (iv) combined paclitaxel + ramucirumab (*n* = 11), (v) combined S-1 + cisplatin (*n* = 8), (vi) combined S-1 + docetaxel (*n* = 7), (vii) combined capecitabine + oxaliplatin (*n* = 5), (viii) combined S-1 + oxaliplatin (*n* = 4), (ix) combined capecitabine + cisplatin (*n* = 3), (x) combined S-1 + paclitaxel + intraperitoneally infused paclitaxel (*n* = 3), (xi) combined CPT-11 + cisplatin (*n* = 2), and (xii) combined capecitabine + trastuzumab (*n* = 1). Of the 67 patients who underwent chemotherapy for RGC, 55 patients (82.1%) received second-line chemotherapy.

### Definition of side effect

The side effects of chemotherapy were graded according to the National Cancer Institute Common Toxicity Criteria, version 4.0 [29]. In this study, we examined the side effects observed during cycles 1–2 of first-line chemotherapy. If multiple side effects were observed, the higher grade was used in the present study. We specifically focused on haematologic toxicity (neutropenia, thrombocytopenia, and anaemia), febrile neutropenia (FN), and gastrointestinal toxicity (diarrhoea, vomiting, and stomatitis).

### Statistical analysis

Continuous variables were expressed as means ± standard deviation and compared using the Mann–Whitney U test. The χ^2^ test or Fisher’s exact test was used to compare categorical variables. Survival curves were calculated using the Kaplan–Meier method, and differences between survival curves were examined using the log-rank test. The univariate and multivariate analyses of the prognostic factors of overall survival (OS) were performed using Cox’s proportional hazards model. *P* < 0.05 was considered significant, and all statistical analyses were performed using SPSS software (SPSS for Windows version 24; IBM Corp., Armonk, NY, USA).

## Results

### Patient characteristics

The average SMI was 42.3 cm^2^/m^2^, and the median SMI for each sex (male, 43.9 cm^2^/m^2^; female, 34.7 cm^2^/m^2^) was used as cutoff values to classify patients into a high SMI group (SMI^High^ group; *n* = 34) and a low SMI group (SMI^Low^ group; *n* = 33). The patients’ clinicopathological characteristics are shown in Table 1. Overall, there were 55 male and 12 female patients, and their average age was 67.6 years. The Eastern Cooperative Oncology Group performance status (ECOG PS) of 59 and 8 patients was 0–1 and 2, respectively. Details of the initial surgery showed that 8 patients had stage I disease, 20 had stage II disease, and 39 had stage III disease. The most common metastatic site was the peritoneum, followed by the haematogenous site and lymph nodes. Regarding histology, 36 patients had a differentiated-type carcinoma and 31 had an undifferentiated-type carcinoma. The relationships between SMI and clinicopathological variables of the patients are shown in Table 1, and the relationships between SMI and characteristics of initial surgery of the patients are shown in Table 2. Body mass index (BMI) was significantly higher in patients in the SMI^High^ group than in the SMI^Low^ group (*P* < 0.001). The number of chemotherapy lines was significantly lower in the SMI^Low^ group than in the SMI^High^ group (*P* = 0.049). No significant differences were observed with respect to age, sex, ECOG PS, adjuvant chemotherapy, prognostic nutritional index (PNI), metastatic site, tumour size, tumour invasion depth, lymph node metastasis, histological type, lymphatic invasion, venous invasion, pathological stage, tumour size, and gastrectomy type.

**Table 1:** Clinicopathological characteristics of SMI^High^ and SMI^Low^ RGC patients

	All patients (*n* = 67)	SMI^High^ (*n* = 34)	SMI^Low^ (*n* = 33)	*p* value
Age (years)	67.6 ± 9.9	66.7 ± 9.7	68.6 ± 10.1	0.376
Sex				0.954
Male	55 (82.1)	28 (82.4)	27 (81.8)	
Female	12 (17.9)	6 (17.6)	6 (18.2)	
ECOG PS (0/1/2)				0.121
0, 1	59 (88.1)	32 (94.1)	27 (81.8)	
2	8 (11.9)	2 (5.9)	6 (18.2)	
BMI	19.4 ± 3.0	20.9 ± 3.0	17.9 ± 2.0	< 0.001
SMI	42.3 ± 7.7	47.4 ± 6.5	37.1 ± 4.6	< 0.001
Adjuvant chemotherapy				0.140
Present	50 (74.6)	28 (82.4)	22 (66.7)	
Absent	17 (25.4)	6 (17.6)	11 (33.3)	
Number of chemotherapy line				
One	12 (17.9)	3 (8.8)	9 (27.3)	0.049
Two and more	55 (82.1)	31 (91.2)	24 (72.7)	
PNI	45.9 ± 7.7	47.0 ± 5.7	44.7 ± 7.7	0.241
Peritoneum metastasis				0.715
Positive	33 (49.3)	16 (47.1)	17 (51.5)	
Negative	34 (50.7)	18 (52.9)	16 (48.5)	
Haematogenous metastasis				0.545
Positive	22 (32.8)	10 (29.4)	12 (36.4)	
Negative	45 (67.2)	24 (70.6)	21 (63.6)	
Lymph node metastasis				0.856
Positive	21 (31.3)	11 (32.4)	10 (30.3)	
Negative	46 (68.7)	23 (67.6)	23 (69.7)	

Data are presented as the mean ± standard deviation or number (percentage) of patients

*BMI* body mass index, *PNI* the prognostic nutritional index, *RGC* recurrent gastric cancer, *SMI* skeletal muscle mass, *SMI*^*High*^ high skeletal muscle mass, *SMI*^*Low*^ low skeletal muscle mass

**Table 2:** Clinicopathological characteristics at initial surgery in SMI^High^ and SMI^Low^ RGC patients

	All patients (*n* = 67)	SMI^High^ (*n* = 34)	SMI^Low^ (*n* = 33)	*p* value
Depth of tumour invasion				0.846
T1	10 (14.9)	4 (11.8)	6 (18.2)	
T2	8 (11.9)	4 (11.8)	4 (12.1)	
T3	28 (41.8)	14 (41.1)	14 (42.4)	
T4	21 (31.4)	12 (35.3)	9 (27.3)	
Lymph node metastasis				0.461
Positive	57 (85.1)	30 (88.2)	27 (81.8)	
Negative	10 (14.9)	4 (11.8)	6 (18.2)	
Histologic type				0.895
Differentiated	36 (53.7)	18 (52.9)	18 (54.5)	
Undifferentiated	31 (46.3)	16 (47.1)	15 (45.5)	
Lymphatic invasion				0.537
Positive	64 (95.5)	33 (97.1)	31 (93.9)	
Negative	3 (4.5)	1 (2.9)	2 (6.1)	
Venous invasion				0.371
Positive	61 (91.0)	32 (94.1)	29 (87.9)	
Negative	6 (9.0)	2 (5.9)	4 (12.1)	
Stage of disease				0.281
I	8 (11.9)	3 (8.8)	5 (15.2)	
II	20 (29.9)	8 (23.5)	12 (36.4)	
III	39 (58.2)	23 (67.7)	16 (48.4)	
Tumour size (mm)	10.0 ± 2.0	12.7 ± 25.8	7.3 ± 11.0	0.072
Type of gastrectomy				0.478
Distal	30 (44.8)	13 (38.2)	17 (51.5)	
Proximal	6 (9.0)	4 (11.8)	2 (6.1)	
Total	31 (46.2)	17 (50.0)	14 (42.4)	

Data are presented as number (percentage) of patients

*RGC* recurrent gastric cancer, *SMI* skeletal muscle mass, *SMI*^*High*^ high skeletal muscle mass, *SMI*^*Low*^ low skeletal muscle mass

### Details of first-line chemotherapy and side effects

**Fig. 2:**
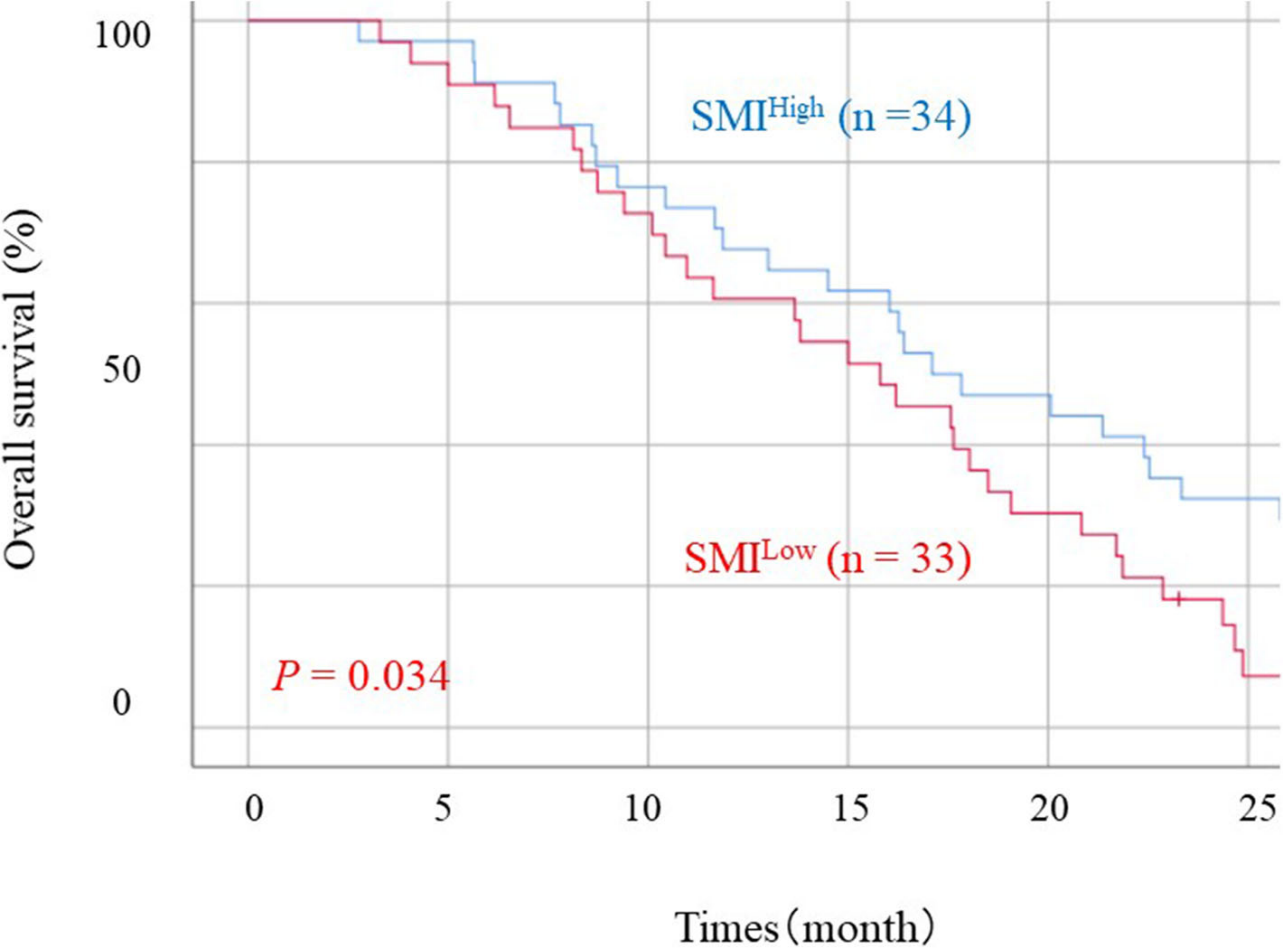
Overall survival curves according to SMI in patients with RGC. Abbreviations: RGC, recurrent gastric cancer; SMI^High^, high skeletal muscle mass; SMI^Low^, low skeletal muscle mass

The details of SMI and first-line chemotherapy are presented in Table 3. The SMI^Low^ group included more patients treated with monotherapy compared with the SMI^High^ group (*P* = 0.016). The details of SMI and side effects are presented in Table 4. The incidence of all side effects of grade 3 or 4 was significantly higher in the SMI^Low^ group than in the SMI^High^ group (*P* = 0.010). The incidence of grade 3 or 4 gastrointestinal toxicity was significantly higher in the SMI^Low^ group than in the SMI^High^ group (*P* = 0.018). No significant differences were observed in terms of neutropenia, anaemia, thrombocytopenia, and FN. In patients receiving monotherapy, the incidence of grade 3 or 4 gastrointestinal toxicity tended to be higher in the SMI^Low^ group than in the SMI^High^ group (*P* = 0.106, Table 5). In patients receiving combined chemotherapy, the incidence of grade 3 or 4 gastrointestinal toxicity was significantly higher in the SMI^Low^ group than in the SMI^High^ group (*P* = 0.011, Table 5). The median survival rate was significantly higher in the SMI^High^ group than in the SMI^Low^ group (17.8 vs 15.8 months; *P* = 0.034, Fig. 2).

**Table 3:** The detail of first-line chemotherapy between RGC patients with SMI^High^ and those with SMI^Low^

	SMI^High^ (*n* = 34)	SMI^Low^ (*n* = 33)	*p* value
Monotherapy	**7 (20.6)**	**16 (48.5)**	0.016
CPT-11	4 (11.8)	7 (21.2)	
S-1	1 (2.9)	7 (21.2)	
Paclitaxel	2 (5.9)	2 (6.1)	
Combined chemotherapy	**27 (79.4)**	**17 (51.5)**	
Paclitaxel + ramucirumab	6 (17.7)	5 (15.1)	
S-1 + docetaxel	6 (17.7)	2 (6.1)	
S-1 + cisplatin	5 (14.7)	2 (6.1)	
Capecitabine + oxaliplatin	1 (2.9)	4 (12.1)	
S-1 + oxaliplatin	4 (11.8)	0	
Capecitabine + cisplatin	1 (2.9)	2 (6.1)	
Combined S-1 + paclitaxel + intraperitoneally infused paclitaxel	2 (5.9)	1 (3.0)	
CPT-11 + cisplatin	1 (2.9)	1 (3.0)	
Capecitabine + trastuzumab	1 (2.9)	0	

Data are presented as number (percentage) of patients

*RGC* recurrent gastric cancer, *SMI* skeletal muscle mass, *SMI*^*High*^ high skeletal muscle mass, *SMI*^*Low*^ low skeletal muscle mass

**Table 4:** The detail of side effect between RGC patients with SMI^High^ and those with SMI^Low^

	SMI^High^ (*n* = 34)	SMI^Low^ (*n* = 33)	*p* value
All side effect of grade 3 or 4			0.010
Positive	11 (32.4)	21 (63.6)	
Negative	23 (67.6)	12 (36.4)	
Neutropenia of grade 3 or 4			0.267
Positive	10 (29.4)	14 (42.4)	
Negative	24 (70.6)	19 (57.6)	
Anaemia of grade 3 or 4			0.975
Positive	2 (5.9)	2 (6.1)	
Negative	32 (94.1)	31 (93.9)	
Thrombocytopenia of grade 3 or 4			0.968
Positive	1 (2.9)	2 (6.1)	
Negative	33 (97.1)	31 (93.9)	
Gastrointestinal toxicity			0.018
Positive	2 (5.9)	9 (27.3)	
Negative	32 (94.1)	24 (72.7)	
FN			0.215
Positive	2 (5.9)	5 (15.2)	
Negative	32 (94.1)	28 (84.8)	

Data are presented as number (percentage) of patients

*RGC* recurrent gastric cancer, *SMI* skeletal muscle mass, *SMI*^*High*^ high skeletal muscle mass, *SMI*^*Low*^ low skeletal muscle mass

**Table 5:** The side effects of monotherapy and combined therapy between RGC patients in SMI^High^ and SMI^Low^ groups

Monotherapy	SMI^High^ (*n* = 7)	SMI^Low^ (*n* = 16)	*p* value	Combined chemotherapy	SMI^High^ (*n* = 27)	SMI^Low^ (*n* = 17)	*p* value
All side effects of grade 3 or 4			0.106	All side effects of grade 3 or 4			0.011
Positive	1 (14.3)	8 (50.0)		Positive	10 (37.0)	13 (76.5)	
Negative	6 (85.7)	8 (50.0)		Negative	17 (63.0)	4 (23.5)	

Data are presented as number (percentage) of patients

*RGC* recurrent gastric cancer, *SMI* skeletal muscle mass, *SMI*^*High*^ high skeletal muscle mass, *SMI*^*Low*^ low skeletal muscle mass

### Univariate and multivariate analyses of patients with RGC

We performed a univariate analysis of the clinicopathological factors considered prognostic for OS in patients with RGC. In the univariate analysis, BMI, SMI, histological type, and PNI were identified as prognostic indicators (Table 6). In the multivariate analysis, we included significant parameters that were identified in the univariate analysis. The multivariate analysis revealed that SMI and histological type were independent prognostic factors (Table 6).

**Table 6:** Univariate and multivariate analyses of prognostic factors for OS in RGC patients

	Univariate analysis			Mutivariate analysis		
	Hazard ratio	95% CI	*P* value	Hazard ratio	95% CI	*P* value
Age (≧75 vs <75)	0.966	0.557–1.675	0.903			
Sex (female vs male)	1.084	0.573–2.052	0.803			
ECOG PS (2 vs 0, 1)	1.542	0.929–2.560	0.094			
BMI (< 18.5 vs ≧18.5)	1.859	1.101–3.132	0.020	1.142	0.597–2.184	0.688
SMI (low vs high)	1.744	1.035–2.939	0.037	1.938	1.041–3.608	0.037
pT (4 vs 1, 2, 3)	1.281	0.752–2.181	0.362			
pN (2, 3 vs 1, 2)	1.310	0.775–2.211	0.313			
Lymphatic invasion (2, 3 vs 0, 1)	1.739	0.964–3.133	0.066			
Venous invasion (2, 3 vs 0, 1)	1.266	0.759–2.110	0.367			
Histologic type (undifferentiated vs differentiated)	2.140	1.272–3.601	0.004	1.860	1.067–3.241	0.028
Type of gastrectomy (proximal/distal vs total)	0.885	0.538–1455	0.629			
Adjuvant chemotherapy (present vs absent)	0.793	0.453–1.388	0.417			
PNI (< 44.0 vs ≧44.0)	1.892	1.119–3.198	0.017	1.720	0.895–3.305	0.104
Peritoneum recurrence (present vs absent)	1.414	0.831–2.405	0.202			
Lymph node recurrence (present vs absent)	0.970	0.568–1.657	0.912			
Haematogenous metastasis (present vs absent)	1.000	0.606–1.649	0.999			

*BMI* body mass index, *ECOG PS* Eastern Cooperative Oncology Group performance status, *OS* overall survival, *PNI* the prognostic nutritional index, *pN* pathological lymph node metastasis, *pT* pathological depth of tumour invasion, *RGC* recurrent gastric cancer, *SMI* skeletal muscle mass, *SMI*^*High*^ high skeletal muscle mass, *SMI*^*Low*^ low skeletal muscle mass

## Discussion

In this study, the SMI^Low^ group had significantly more grade 3 or 4 side effects than the SMI^High^ group, even though the SMI^Low^ group underwent less monotherapy than the SMI^High^ group. The SMI^Low^ group had a significantly worse prognosis and significantly less conversion to second-line chemotherapy than the SMI^High^ group.

Moreover, SMI^Low^ in patients with RGC was associated with grade 3 or 4 gastrointestinal side effects; this result was similar to that of a previous report by Carla et al. [30], where the side effects in 55 patients with metastatic breast cancer receiving capecitabine treatment were retrospectively reviewed. The cross-sectional skeletal muscle area at the third lumbar vertebra was measured using CT, and sarcopenia was defined using a previously published cutoff point. Consequently, sarcopenia was found to be associated with grade 3–4 diarrhoea and stomatitis. Shachar et al. also reported that they retrospectively reviewed side effects in 40 patients with metastatic breast cancer and showed that SMI^Low^ was associated with grade 3–4 toxicity [31]. Likewise, Tan et al. reported that sarcopenia was associated with dose-limiting toxicity in 89 patients with oesophagogastric cancer undergoing neoadjuvant chemotherapy [32]. Their multivariate analysis revealed that only sarcopenia was an independent risk factor of dose-limiting toxicity. These results show that SMI^Low^ is related to the high-grade toxicity of chemotherapy. However, the mechanism associating SMI^Low^ and toxicity is unclear. One possible explanation is that changes in body composition are related to alterations in the distribution and clearance of the anticancer agent [32]. 5-FU, a key drug in gastric cancer, is hydrophilic but widely distributed through active transport [33]. This drug undergoes extensive metabolism, primarily through dihydropyrimidine dehydrogenase. Variants of dihydropyrimidine dehydrogenase have been associated with an increased risk of 5-FU toxicity [34]. The clearance of 5-FU is increased in individuals with higher SMM [35]. These findings suggest that decreased clearance of 5-FU due to SMI^Low^ may be related to the increased side effects. To the best of our knowledge, this is the first report in which SMI^Low^ was associated with grade 3 or 4 side effects in patients with RGC.

Sarcopenia has been shown to negatively impact long-term outcomes of patients with several cancer types [11, 36, 37]. However, only a few studies have investigated the effect of sarcopenia on the prognosis of patients with RGC. Willemieke et al. retrospectively reviewed the prognosis of 88 patients with advanced oesophagogastric cancer treated with standard first-line palliative chemotherapy. They showed that the survival rate was not different between patients with and without sarcopenia in univariate and multivariate analyses. The cutoff in this study was set based on the presence or absence of sarcopenia, which is different from our cutoff, and may have caused the discrepancy. By contrast, Kouzu et al. concluded that sarcopenia was a poor prognostic factor after gastric cancer recurrence. They retrospectively reviewed 67 patients who experienced gastric cancer recurrence and found that sarcopenia was an independent negative prognostic factor in a multivariate analysis, which is similar to our result. They calculated the psoas muscle index (PMI) and used a receiver operating characteristic curve to determine the cutoff PMI. These results suggest that SMI^Low^ may be associated with prognosis, although there are problems with SMI evaluation and setting of the cutoff values.

The reason for the poor prognosis of patients with SMI^Low^ RGC has not been sufficiently elucidated. One potential explanation is that these patients were less likely to receive second-line chemotherapy. The first choice of treatment for patients with metastatic gastric cancer is chemotherapy, and patients need to undergo second- and third-line chemotherapy for further improvement of treatment outcomes [38–40]. In this study, the rate of second-line chemotherapy was significantly lower in the SMI^Low^ group than in the SMI^High^ group, which might have led to the poor prognosis in the SMI^Low^ group.

The standard first-line palliative systemic chemotherapy is a combined regimen in accordance with the Japanese gastric cancer treatment guidelines [41]. However, SMI^Low^ in patients with RGC was associated with grade 3 or 4 side effects and the rate of second-line chemotherapy was significantly lower in the SMI^Low^ group than in the SMI^High^ group. Gastrectomy causes not only weight loss but also SMM loss [21]. Preoperative nutritional and exercise interventions for gastric cancer may be useful in improving postoperative outcomes [42]. However, few reports have focused on the effects of postoperative nutritional or rehabilitative interventions on the postoperative development of sarcopenia and outcomes [43]. Postoperative nutritional management and regular exercise may be important for the maintenance of SMM and nutritional status at the time of gastric cancer recurrence in patients at a high risk of recurrence after gastrectomy. In addition, nutritional and rehabilitative interventions during chemotherapy to maintain SMM and nutritional status may be associated with decreased side effects and an increased rate of second-line chemotherapy administration.

This study has several limitations. First, we conducted this study in a single institution, and the number of patients who experienced postoperative recurrence was relatively small. Second, the optimal cutoff SMI value has not been determined in patients with RGC. Because postoperative patients with gastric cancer often have lower dietary intake, body weight, and SMM, and reports of SMM in patients with RGC are few, hence, the SMI median value of all patients was used as the cutoff value in this study. Third, the first-line chemotherapy was not unified. Although this was a long-term study and the guidelines changed over time, chemotherapy was administered in accordance with the guidelines at that time. Therefore, well-designed, randomised, prospective studies with larger populations are needed to confirm these findings.

In conclusion, patients with SMI^Low^ RGC had significantly more grade 3 or 4 side effects than those with SMI^High^, and SMI was a useful prognostic marker of RGC. In patients with advanced gastric cancer after gastrectomy with a high risk of recurrence, interventions to prevent the loss of SMM, such as nutritional therapy and regular exercise, might be needed to improve the prognosis in patients with RGC.

## Data Availability

The datasets used and analysed during the current study are available from the corresponding author on reasonable request.

## Abbreviations

BMI: Body mass index
ECOG PS: Eastern Cooperative Oncology Group performance status
FN: Febrile neutropenia
OS: Overall survival
PNI: The prognostic nutritional index
pN: Pathological lymph node metastasis
pStage: Pathological stage
pT: Pathological depth of tumour invasion
RGC: Recurrent gastric cancer
SMI: Skeletal muscle mass index
SMI^High^: High skeletal muscle mass
SMI^Low^: Low skeletal muscle mass
SMM: Skeletal muscle mass
